# Gold Foil

**Published:** 1869-12

**Authors:** G. V. Black


### SELECTED ARTICLES.

### ARTICLE VII.

### *Gold Foil.*

By Dr. G. V. Black.

(*Concluded.*)

*Forms of gold.*—A few words may be said of the manner of handling and forming the pieces of gold. This may be stated as a positive rule to which there should be no exception: that gold foil, the welding property of which is to be made use of, should never be touched with bare fingers, for the reason that the exhalations from the skin always injure this property more or less. The gold is easiest and most perfectly worked with the fingers neatly gloved with fine chamois skin or linen. These gloves should not remain on the hand any longer than is actually necessary to do the work, and should be very carefully kept. It may be thought that it would be inconvenient to handle the gold in them, but this soon wears away with practice, and becomes easier and the results more satisfactory than with any of the gold crumpers that I have seen. I notice some ingenious Yankee has gotten up a patent instrument for rolling up the ropes, which consists of two boards with a strip of rubber stretched between. This may produce a very nice rope, but after studying the effects of rubber on gold, I should not prefer it to the fingers. We should remember that it is not moisture merely that injures our gold, for that is driven off by annealing; but it is the gases and solid residue from these exhalations, compared with which the sulphurous acid exhaled on rubbing rubber is no improvement. I would kindly suggest that he substitute a nice piece of chamois in place of the rubber.

*The Block.*—This form is best made by taking the amount of gold wanted in a single piece, and crumpling it loosely together, and then bringing it into a square form with a pair of flat-bladed thumb pliers. The whole operation should be conducted in such a manner that the finished block will be of equal density thoughout.

*Ovoid Pellets* are made in the same manner as the block, except that they are rolled to the desired size and shape—round or oblong—with the gloved fingers.

*The Rope* is made by folding a sheet or part of a sheet of gold loosely together into a long rouud roll, and then twisting it into a moderately compact rope. It is also made by rolling the gold in a napkin, and by crumpers, but much the best form is produced by the fingers gloved with chamois skin or linen.

*The Pellet* is produced by cutting the *Rope* into short lengths. I am using a pair of shears prepared with a graduated scale attached to one of the blades, with which the length of each pellet is definitely and easily measured as it is cut, without any loss of time whatever. This is a very convenient and useful instrument to those who may wish to adopt definite sizes of the pellet.

*The Ribbon* is best produced by using two prism-shaped blocks of convenient lengths, (one side about half the length of each of the others is the most convenient form.) A paper knife or ivory spatula may be used instead of the second block. The gold is laid on a piece of velvet, the thin edge of one of the blocks placed on its centre—the second block is now passed under the edge of the sheet, which is folded over the first, which is withdrawn, and the gold brought down smoothly by passing the block over it. This operation is repeated until the desired width is obtained, and the ribbon is ready for use. I know of no other mode of producing ribbon which can compare with this for perfection or ease to the operator.

*The Cylinder* is made by rolling a ribbon smoothly and compactly upon a broach or four-sided drill, as a ribbon is rolled upon a block, then by rolling the broach or drill backward it is loosened, and the cylinder removed. The width of the ribbon should equal the desired length of the cylinder. Many other forms have been used, more or less, but they are generally modifications of those given, and hardly require separate consideration.

The form in which gold foil may best be used in filling teeth, is a question of no small importance, and deserves much careful consideration. We want that form which may best be impacted securely and perfectly into grooves and retaining pits, and against the walls of the cavity. Compared with these points solidity itself is a secondary consideration. If our form of foil should not admit of the ready accomplishment of these objects, our whole work is liable to fail, no matter how solid other parts of the filling may be, or how beautiful it may appear. To meet these requirements our form of gold should be very impressible, and take readily the form of the wall of the cavity against which it may be impacted. These requirements seem to be most perfectly met by the form of the block. This form of gold is soft and sponge-like, may be pulled this way or pushed that way with the plugger, and adapts itself most perfectly to the walls of the cavity, but has the objection that it requires more labor to consolidate it perfectly than most other forms on account of the many wrinkles and sharp angles which must be beaten out. This objection is perhaps much greater in theory than in actual practice, and whether it is or not, the impaction against the walls is of much more importance, as before stated, than the actual solidity of the body of the work; provided always, that the solidity is sufficient for the ordinary wear and tear of mastication, which is easily attained with these blocks. The working of the ovoid pellet is about the same as the block. The next form nearest resembling the block is the pellet. This form is not crumpled quite so much as the block, yet is almost as soft,and is well adapted into working into small cavities or retaining pits, grooves and undercuts. The use of pellets cut from ropes larger than those made from a single sheet of No. 4 gold is objectionable, onaccount of the condensation which occurs at the ends of the pellets in cutting them. The ease and rapidity with which they may be prepared is quite an item in their favor, in all cases where small pieces are needed. The rope has been much used in times past by beginning with one end and folding it into the cavity, condensing each fold as it is laid in. I think this practice is mostly abandoned on account of the obvious difficulty of keeping the rope free from the moisture of the mouth, and that it obscures the cavity too much.

The cylinder is a very popular form of gold with many operators, and seems to have been gaining ground for a number of years. It is used by setting it on end in the cavity to be filled,and condensing against one of its walls, or if it be an approximal cavity, by laying the end of the cylinder out over the wall. With this form of gold there are no angles to break down in condensing, so that the solidity is attained with less labor than with most other forms in use. In fact, the gold is placed in the cavity in a very smooth and compact state, so that little more condensation is required, and is generally used in pretty large masses, particularly if the cavity be large. It is not so easily impacted into any irregularities which may occur in the walls of the cavity, although in the main very great solidity is attained. At those points along the wall where two cylinders lie against each other, triangular openings are liable to occur. In case of approximal cavities where the plan is to lay the ends of the cylinders out over the walls, this may result in serious mischief. This may be illustrated by taking three little rolls or cylinders of paper, with square ends, and placing them together, a triangular opening will be observed in the centre; or if you take two of them and press them together against a flat surface, a similar opening will be observed between them. It will be found a very difficult matter to close these openings perfectly and solidly by any practical amount of pressure. If the space be filled at all it is apt to be filled very loosely. The difficulty is not lessened by substituting gold for paper, and I consider it a very grave objection to the general use of cylinders in filling teeth, especially in approximal cavities. I have myself been greatly mortified to find fillings which I had thought very perfect, imbibing moisture causing decay to start, evidently from this cause; and have also noticed it in fillings made by some very fine operators. To overcome this trouble requires considerable manipulative skill, and much good judgement, and even then it seems to be a somewhat dangerous form of gold to use largely.

The ribbon cut in short lengths or folded in as rope, may be used in filling teeth; but is scarcely applicable to the filling of cavities, for the reason that it is too much condensed, and too many hard corners are liable to occur to allow it to be spread and impacted perfectly against the walls of the cavity. This form of gold cut into convenient lengths may be used with great advantage in building up lost parts of teeth, after the cavity has been filled out even with the walls. In such cases the slips may be laid on smoothly, and condensed with less labor than any other form of gold. There seems to be very little attention paid by dentists to the sizes of the pieces of gold used; or, I should say, they do not adopt any regular system of sizes, but prepare their gold according to the size of the cavity to be filled. Now to prepare gold in such a manner as this, seems to me to be forever experimenting with vague and undefined sizes. And we cannot feel that degree of confidence and thorough knowledge of what is coming under our instrument that we should. I would recommend every operator to adopt some regular gradation of sizes which may be found convenient, and adhere to them closely in all their work.

A few weeks' observation will be sufficient to determine upon those sizes which will be most convenient for their range of operations. Then a known and fixed size of gold will be chosen to be worked to a given place as the eye may dictate, and there will be no vagueness as to the working of the piece under the instrument, for it will be perfectly familiar. Those who have not tried this plan will be surprised at the relief it will afford in all difficult operations. Instead of feeling their way along with perhaps some misgivings as to whether the gold prepared is going to suit the situation all the way through, they feel that they have definite and familiar sizes of gold at hand, at once adaptable to any situation ; a view of which is always sufficient to determine the size or form to be taken. The adoption of such a system will be of no small advantage to those writing and speaking upon the subject, in making themselves understood or in conveying a distinct idea of their plans to their brothers; for it is often the case that the size of the gold is as important as the form. We all perhaps feel how indistinctly our idea of the size of gold used in any given case is conveyed in any form of words used by the profession. We need some system by which an accurate idea of this kind may be easily expressed and generally understood. Then much of the vagueness which hangs around our operations when put in the form of words, would disappear. But such a state of things cannot possibly occur when operators cannot tell in words what size the pieces of gold used in such and such an operation should be. The plan which I have adopted, and which, perhaps, is as simple and as easily understood by all as any other, is that of naming each piece according to the number of grains, or the fraction of a grain contained in a piece. Thus, if a sheet of No. 4 foil be cut into four equal parts, and each part made into a block, they will be called one grain blocks ; if it be divided into eight equal parts, they will be half grain pieces. No. 2 gold divided as the last will make one-fourth grain pieces. A sheet of No. 4 gold would make a four grain block or cylinder ; a half sheet would make a two grain piece, and so on throughout all sizes that may be wanted. One number of foil is adapted to this system as well as another, and it is applied to all forms of gold alike. This plan presents an unlimited variety of sizes a very few of which, when properly selected, will be found sufficient for any operation. Any one using any selection of these will understand readily what is meant when any one of them may be mentioned, whether they be sizes he is in the habit of using or not.

I am using half a dozen of these in my office for general use; 1 grain, $\frac{2}{3}$ grain and $\frac{1}{2}$ grain in the form of blocks, and $\frac{1}{3}$, $\frac{1}{8}$ and $\frac{1}{4}$ of a grain in the form of a pellet, and occasionally some of larger or smaller size. But in whatever form my gold is prepared, it always takes these definite proportions.

I find this system very convenient in directing my assistant in the gold I may want; and, in fact, it is a convenience in every way you may put it.

The preparation of gold I make entirely the work of my assistant. She has her chamois skin gloves and all the necessary instruments in a convenient drawer, and, whatever else goes wrong, every piece of gold that comes to the tray to be used must be just right. And I have no hesitation in saying that it may be done as well, and often better, by a properly instructed assistant, than the operator would do it for himself. And more than this, I have my assistant do all my annealing. For this purpose she is supplied with some annealing forks, which are made by flattening an old excavator, splitting it up with a separating file, and sharpening the points. This will stick into any piece of gold, and hold it without condensing it, and is preferable to the foil pliers in general use. With one of these instruments she takes such a number and form as I may call for, passes it through the alcohol flame, and then into the cavity to be filled, when I take it with my plugger. And while I may be fixing it for the final condensation, she has taken up and annealed another piece, which is held while the first is condensed with the mallet, she always bringing the number called, and annealing lightly or otherwise, as may be directed. The position each should occupy in the cavity is pointed out by a slight motion of the point of the plugger over the spot.

I find this plan to work smoothly and well, and saves much valuable time in the annealing of the gold.

We started out with our gold foil at the time when it was received in good condition from the beater or dealer. We have been with it through that often dangerous interval which has been spent in the gold drawer, awaiting quietly its work of protection to some once beautiful and pearly organ of mastication, undergoing a slow destruction by the unrelenting destroyer. We have kept watch as well as our feeble powers would admit, for those intruders which might endanger its usefulness, and have done our best to baffle their designs. We have seen the time roll round when it has been called to duty, and have watched anxiously while it has been made to take the forms best adapted to the peculiar duty assigned it, and have at last seen it nestled away solidly and snugly in the home invitingly made for it by the dispossessed monster, DENTAL CARIES.—*Missouri Dent. Jour.*

